# ZrN Phase Formation, Hardening and Nitrogen Diffusion Kinetics in Plasma Nitrided Zircaloy-4

**DOI:** 10.3390/ma14133572

**Published:** 2021-06-25

**Authors:** Robert Balerio, Hyosim Kim, Andres Morell-Pacheco, Laura Hawkins, Ching-Heng Shiau, Lin Shao

**Affiliations:** 1 Department of Nuclear Engineering, Texas A&M University, College Station, TX 77843, USA; baleriosite@tamu.edu (R.B.); anmorell@tamu.edu (A.M.-P.); lhawk1124@yahoo.com (L.H.); jackhern@tamu.edu (C.-H.S.); 2 MST-8, Los Alamos National Laboratory, Los Alamos, NM 87545, USA; hkim@lanl.gov

**Keywords:** nitridation, Zircaloy, indentation, nitrogen diffusion

## Abstract

Plasma nitridation was conducted to modify the surfaces of Zircaloy-4. Scanning electron microscopy (SEM), transmission electron microscopy (TEM), and Raman analysis were used to characterize microstructures and phases. Surface indentation and cross-sectional indentation were performed to evaluate mechanical property changes. Nitridation forms a thin layer of ZrN phase, followed by a much deeper layer affected by nitrogen diffusion. The ZrN phase is confirmed by both TEM and Raman characterization. The Raman peaks of ZrN phase show a temperature dependence. The intensity increases with increasing nitridation temperatures, reaches a maximum at 700 °C, and then decreases at higher temperatures. The ZrN layer appears as continuous small columnar grains. The surface polycrystalline ZrN phase is harder than the bulk by a factor of ~8, and the nitrogen diffusion layer is harder by a factor of ~2–5. The activation energy of nitrogen diffusion was measured to be 2.88 eV. The thickness of the nitrogen-hardened layer is controllable by changing the nitridation temperature and duration.

## 1. Introduction

Zirconium-based alloys are widely used as fuel cladding and in-core structural components in light water reactors due to their low neutron absorption cross-section, good corrosion resistance, and high strength [1]. The advantages of Zr fuel cladding are countered by its anisotropic properties and poor oxidation response in steam at high temperatures. While Zr cladding exhibits high corrosion resistance under normal operating conditions, its vulnerability to hydrogen pickups and rapid oxidation are well-known issues in the event of a loss of coolant accident (LOCA). Reactions between the Zircaloy fuel cladding and water vapor result in abnormal oxidation, and subsequent hydrogen production, as occurred in the Fukushima Daiichi nuclear accidents.

One technique envisioned to avoid the above scenarios is to coat fuel cladding tubes with an oxidation-resistant and thermally stable material [2]. An ideal coating is expected to reduce oxidation rates under abnormal conditions to avoid possible hydrogen explosions when Zircaloy-4 is exposed to super-hot steam. Adhesion of the coating film with tubes and structural and chemical integrity with Zircaloy are highly demanded. Currently, coating candidates include FeCrAl [3], Cr [4], and Al-containing MAX (M_n + 1_AX_n_, M = early transition metal, A = group-A element, X = C or N, n = 1–3) phase [5]. However, the easy swelling of Cr at relatively low damage levels [6], each amorphization of MAX phase alloys (Ti_2_AlC as one example) at interfaces [7], and complicated interaction reactions between FeCrAl and Zircaloy require further investigation and optimization [8]. Hence, other coating alternatives are still required.

Of the various coating approaches, including cold spray coating [9], chemical vapor deposition [10], and laser coating [11], nitrogen-based plasma nitridation is a unique approach to coating a nitride layer on a metal surface with the following benefits [12]: (1) it is a low-temperature process, which avoids degradation of bulk materials; (2) it converts the surface of the original substrate into a nitride layer, which minimizes the risk of debonding; (3) the process is adjustable to control the thickness of the nitride layer; (4) the process can be optimized to coat a continuous film to introduce nitride particles in the matrix; and (5) the process can be used to treat substrates of complicated geometry.

There are two plasma nitridation approaches. One is direct nitridation, in which a substrate is directly biased and exposed to plasma [12]. The other uses a hollow cage to induce plasma, and a substrate is positioned within the cage [13,14]. Plasma either randomly bombards the substrate when the substrate is floated or directionally diffuses toward the substrate if the substrate is biased. Such cage-assisted plasma treatment provides the benefit of increased uniformity since the cage holes play an important role in determining plasma distribution [12,13,14].

The effects of plasma nitridation on various metals are well-known and have been documented [1]. However, almost nothing is known about the nitridation of Zircaloy-4. We were motivated in the present study to study the feasibility of the technique to modify Zircaloy-4 tubes. In addition to a zirconium nitride layer as a coating option to further increase accident tolerance of fuel cladding, it can be coated on the inner side of a tube as a diffusion barrier to reduce fuel-cladding interactions when significant fuel swelling develops under high burnups. For other coating techniques, coating the inner side of a fuel cladding is almost impossible, but plasma-based techniques are feasible to treat both inside and outside surfaces.

## 2. Materials and Methods

We used a homemade cathodic cage plasma nitriding (CCPN) device. Details of the device were reported elsewhere [15]. The Zircaloy-4 composition was Zr-1.5%Sn-0.2%Fe-0.1%Cr. The reactor-grade Zircaloy-4 was obtained from Westinghouse Electric Company LLC (Cranberry Township, PA, USA). Prior to nitridation, Zircaloy-4 was cut into pieces of 10 mm × 5 mm × 1 mm from a large block, and was polished using 1200 grit SiC paper. Samples were positioned within a cage, which was composed of Zr702. The cage top was 1 mm thick perforated Zr702 with 6.35 mm staggered holes and a 50% open area spacing. The height and diameter of the cage were 30 and 60 mm, respectively. The electrically conductive cage was placed on the base, and a cathodic voltage was applied via the cage base. As a first step, a bias was applied to the cage to induce plasma formation. Zircaloy-4 samples were either biased together with the cage or floated. When floated, samples were expected to receive less plasma flux. Prior to applying a bias, the target chamber was pumped and re-filled with N_2_ (90%)/H_2_ (10%) gas mixture several times. H_2_ gas was used to minimize oxidation. Under sufficiently high bias and with sufficient gas pressure, gas atoms were ionized and created secondary ionization cascades. Plasma was created around the cage holes, which diffused toward Zircaloy-4 samples within the cage. The plasma was ignited at a low bias and then the pressure was slowly increased and stabilized to 200 Pa. The substrate temperatures (from 600 to 840 °C) were controlled by cathode voltages (from about 500 to about 600 V).

After plasma nitridation, samples were characterized by using scanning electron microscopy (SEM), transmission electron microscopy (TEM), Raman, and nanoindentation. The focused ion beam (FIB) technique was used to prepare TEM specimens using a 5–30 keV Ga beam. The 30 keV Ga beam was first used for cutting and lift-out, followed by a 5 keV Ga beam with a weaker beam current for the final stage of specimen thinning to a thickness of ~100 nm. TEM characterization was conducted using a FEI Tecnai F20 ST (FEI Company, Hillsboro, OR, USA) with an operating voltage of 200 kV. For Raman characterization, a Horiba Jobin-Yvon LabRam HR Raman Confocal Microscope (Horiba, Kyoto, Japan) was used. The wavelength of the laser was 633 nm. The laser spot size was 150 µm. The analysis was performed on the surface of the nitrided sample. SEM analysis was performed with a Tescan LYRA-3 Model GMH Focused Ion Beam Microscope (Tescan, Brno, Czech Republic) to evaluate nitrogen layer thickness.

A Bruker Dimension Icon atomic force microscope (AFM, Bruker Corporation, Billerica, MA, SUA) was used to characterize surfaces before and after nitridation. The hardness and reduced modulus mechanical properties were measured with a Hysitron TI 950 Triboindenter (Hysitron, Inc., Eden Prairie, MN, USA). Prior to and after performing indentations, the calibration of the transducer was verified on fused quartz. Cross-sectional nanoindentation was performed with a diamond Berkovich probe and a low load transducer. A trapezoidal load function was used where load and unload times were 5 s, and the hold time at a peak force of 10 mN was 2 s. Cross-sectional nanoindentation was performed on the cross-section of the nitrided sample at various distances from the surface edge. The distances represent the depth beneath the nitrided surface. For surface indentation as planar characterization, load-partial unload indentations were performed using a high load transducer.

Wavelength dispersive spectroscopy (WDS) was performed using a Cameca SXFive electron microscope (CAMECA, Gennevilliers, France) to analyze nitrogen concentration versus depth in nitrided Zircaloy-4. The beam conditions for sample analysis were 15 kV and a 2 μm beam spot size. Two beam currents of 20 and 60 nA were used. The peaks for all elements of interest were counted for 20 s and the background was counted for 10 s.

## 3. Results

### 3.1. Atomic Force Microscopy Characterization

**Figure 1:**
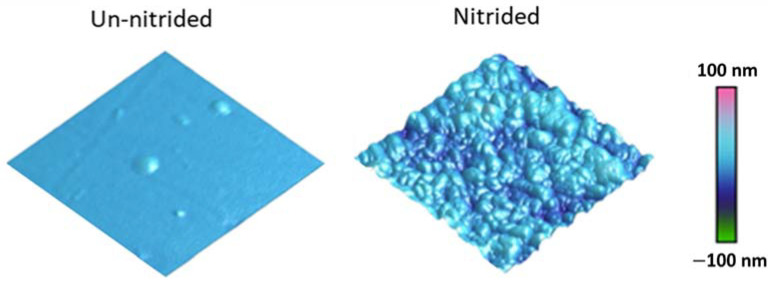
AFM images of Zircaloy-4 with and without nitridation.

Figure 1 compares the AFM images of Zircaloy-4 before (left) and after (right) nitridation at 700 °C. Although the nitrided surface became rough, the roughness changes were still small. The average root mean square roughness (Rq) in the control sample before nitridation was 1.2 nm. After the nitridation, this value increased to 11.8 nm. No precipitations or cracks were observed on the nitrided surface from both AFM images and SEM images (not shown). The surface quality was good enough to allow reliable indentation testing.

### 3.2. Raman Characterization

**Figure 2:**
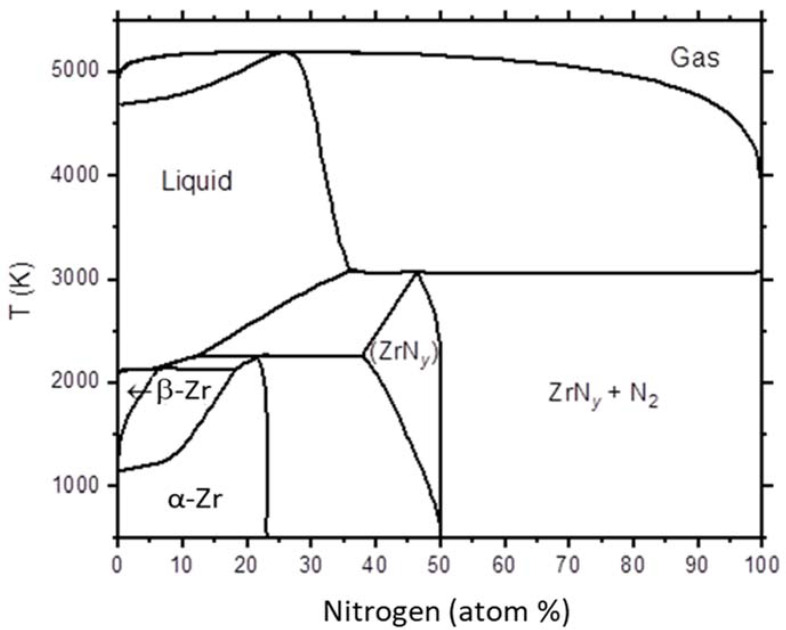
The calculated Zr–N phase diagram. Replotted from reference [16].

Numerous experimental and modeling studies were performed to understand the Zr–N phase diagram [16]. Figure 2 plots one Zr–N phase diagram [1], featured by N-dissolved bcc-Zr (denoted as β-Zr), hcp-Zr (denoted as α-Zr), and a non-stoichiometric fcc structure compound (denoted as ZrN_y_). As shown in Figure 2, at the lowest nitriding temperature of 500 °C, ZrN_0.5_ is expected. At the highest temperature of 800 °C, ZrN_x_ allows a small range of N stoichiometry deviation from ZrN_0.5_. Although it is generally accepted that ZrN is the only stable intermediate compound, many studies have shown another metastable phase Zr_3_N_4_ [17], which may exist as c-Zr_3_N_4_ (Th_3_P_4_ structure) [18] or o-Zr_3_N_4_ (orthorhombic structure) [19,20]. The o-Zr_3_N_4_ was metastable and decomposed into ZrN and N_2_ upon annealing. The formation of ZrN_x_ (NaCl structure) with 1.06 < x < 1.23 was previously claimed [21].

**Figure 3:**
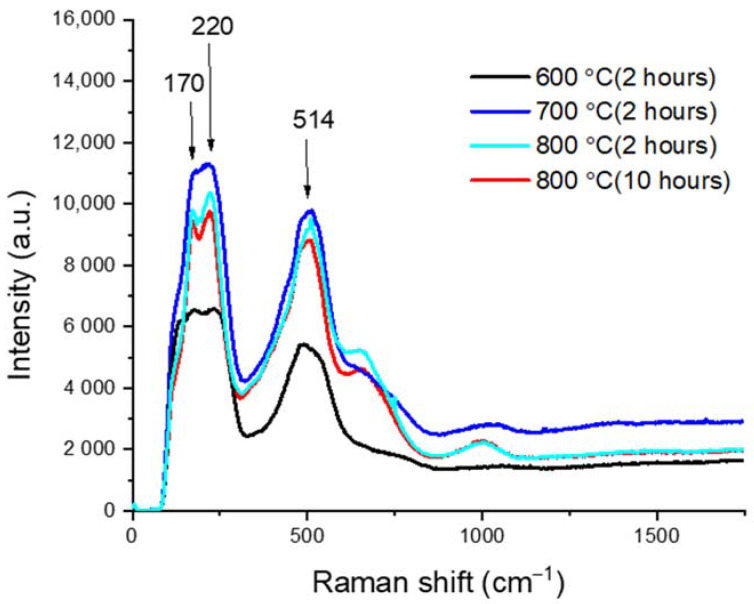
Raman spectra of Zircaloy-4 nitrided at 600, 700, and 800 °C.

Raman characterization confirmed the ZrN phase formation after nitridation. Figure 3 plots the Raman spectra of the nitrided samples at various nitridation temperatures and time duration. All spectra feature multiple peaks at about 170, 220, and 514 cm^−1^. The two peaks at low frequencies, 179 and 232 cm^−1^, are characteristic transverse and longitudinal acoustic bands of ZrN, respectively, due to acoustic phonons [22]. The high-frequency peak at about 500 cm^−1^ is caused by optical phonons in ZrN [22].

### 3.3. Transmission Electron Microscopy Characterization

**Figure 4:**
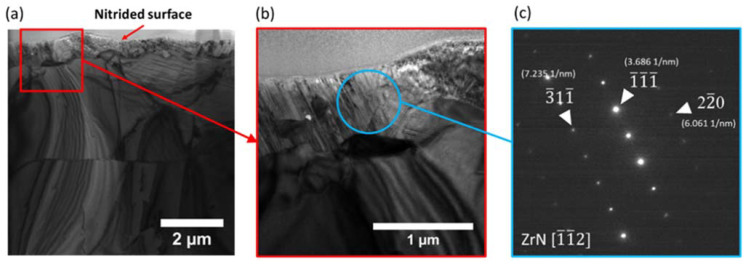
(**a**) Cross-sectional TEM micrograph of nitrided Zr, (**b**) the micrograph of the near surface region marked with a red square in (**a**), and (**c**) localized diffraction pattern obtained from the near surface region marked with a blue circle in (**b**).

**Figure 5:**
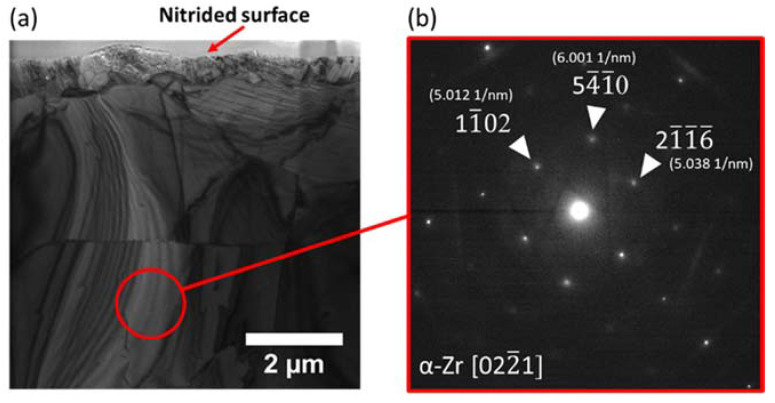
(**a**) Cross-sectional TEM micrograph of nitrided Zr and (**b**) localized diffraction pattern obtained from a region marked with a red circle in (**a**).

Figure 4a,b shows the cross-sectional TEM image of Zircaloy-4 nitrided at 700 °C. In the near-surface region, about 1 μm deep, there is a continuous polycrystalline layer with small columnar like grains. The localized diffraction pattern shows lattice distance matches with [111], [220], and [311] of ZrN phase. Figure 4c is the corresponding localized diffraction pattern. The pattern is indexed, showing good agreement with the ZrN crystal structure. Note that the diffraction pattern disagrees with that of pure Zr and zirconium oxide. Figure 5a shows the cross-sectional TEM image with a red circle highlighting a region a few microns away from the surface. Figure 5b shows the localized diffraction collected from the circled region. The diffraction pattern and lace spacing match that of the α-Zr phase.

### 3.4. Nanoindentation

Nanoindentation tests were performed on both the planar side (indented along the normal direction of the nitrided surface) and the cross-sectional side (indented along the normal direction of the polished cross-section) of nitrided Zircaloy-4. The cross-sectional indentation is used to show the hardness changes as a function of distance from the surface in order to compare the mechanical property changes of the surface nitride layer and the nitrogen diffusion layer. The distance of the indentation spot can be well-controlled, and there is no limitation on the maximum distance. However, the cross-sectional indentation approach does not well-characterize the surface nitride layer due to its spatial resolution. To solve this problem, the traditional surface indentation (indented along the surface normal direction) was used to study the surface nitride layer. Due to its high hardness, the traditional surface indentation approach is limited by its maximum penetration depth. Hence, the combination of these two approaches mitigates the issues and are supplementary to each other.

**Figure 6:**
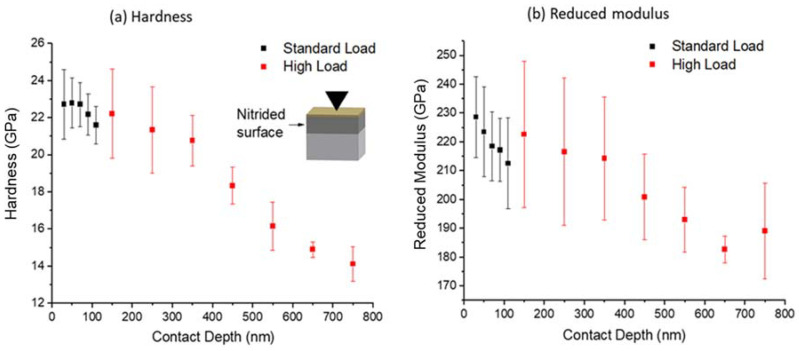
Surface indentation obtained (**a**) hardness and (**b**) reduced modulus as a function of depth in nitrided Zircaloy-4 (nitrided at 800 °C for 24 h). The indentation was applied along the normal direction of the nitrided surface.

**Figure 7:**
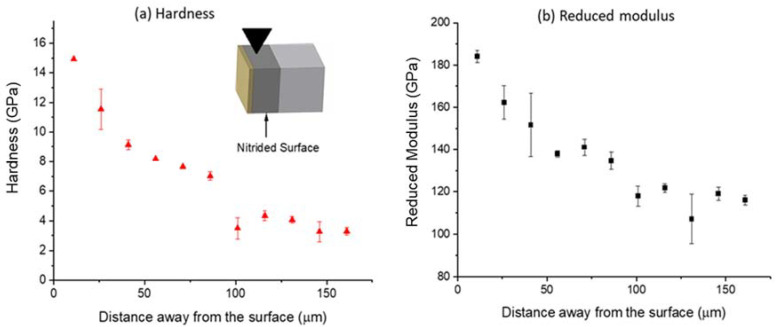
Cross-sectional indentation obtained (**a**) hardness and (**b**) reduced modulus as a function of depth in nitrided Zircaloy-4 (nitrided at 800 °C for 24 h). The indentation was applied along the normal direction of the cross-section of the sample. The depth was determined by the indentation position away from the nitrided surface.

Figure 6 shows hardness (a) and reduced modulus (b) as a function of indentation depth. Note that the indentation was performed on the nitrided surface. The surface layer of about 400 nm is extremely hard, with the peak hardness reaching ~23 GPa. At a depth of about 700 nm, the hardness reduces to about 14 GPa. For reduced modulus, the near-surface region of 400 nm has a value of about ~200 to ~240 GPa. At a depth of 700 nm, the reduced modulus values reduce to about 180 GPa. Figure 7 shows the hardness and reduced modulus obtained from the cross-sectional characterization. Hardness is noticeably higher near the surface to about 100 μm in depth. The hardness values decrease from 15 GPa near the surface to an averaged value of ~7 GPa. At a depth beyond 100 μm, the hardness is about 2 to 4 GPa. The reduced modulus slightly decreases from the highest value of 184 GPa to about 115 GPa at about 100 µm. At a deeper depth, the reduced modulus remains roughly the same.

### 3.5. Nitrogen Profiling

**Figure 8:**
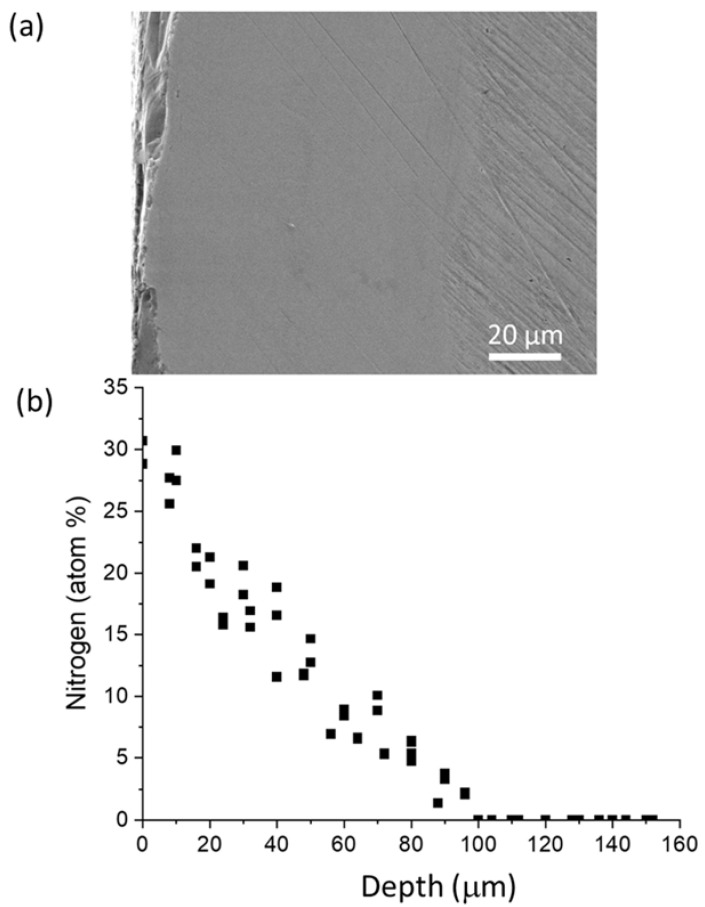
(**a**) SEM image of the polished cross section of Zircaloy-4 after nitridation at 800 °C for 24 h, and (**b**) the corresponding nitrogen profile obtained from cross-sectional WDS.

Hardness changes are closely related to nitrogen distributions. Figure 8 compares the cross-sectional SEM image (Figure 8a) of the polished sample and the nitrogen profile (Figure 8b) obtained from WDS. The sample was nitrided at 800 °C for 24 h. The nitrogen diffusion tail ends at a depth of about 100 µm, which is consistent with the cross-sectional hardness changes, which shows a noticeable hardness drop at a depth ~100 µm (as shown in Figure 8). The SEM image shows that the surface layer from depth 0 to about 100 µm has a relatively smooth surface in comparison with the region deeper than 100 µm. This is also consistent with the hardness changes, since lower hardness leads to deeper scratches after the polishing. The SEM image in Figure 8 shows that the nitrided surface after cross-sectional polishing is quite rough. The roughness occurred from the cross-section cutting instead of the nitridation.

### 3.6. Nitrogen Diffusion Kinetics

**Figure 9:**
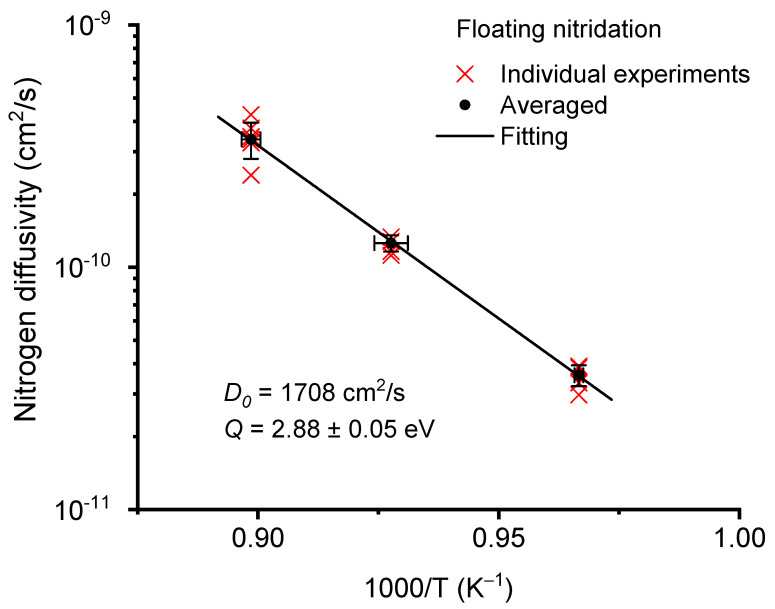
Arrhenius plot of nitrogen diffusivity in Zircaloy-4 as a function of nitridation temperatures for floating nitridation.

**Figure 10:**
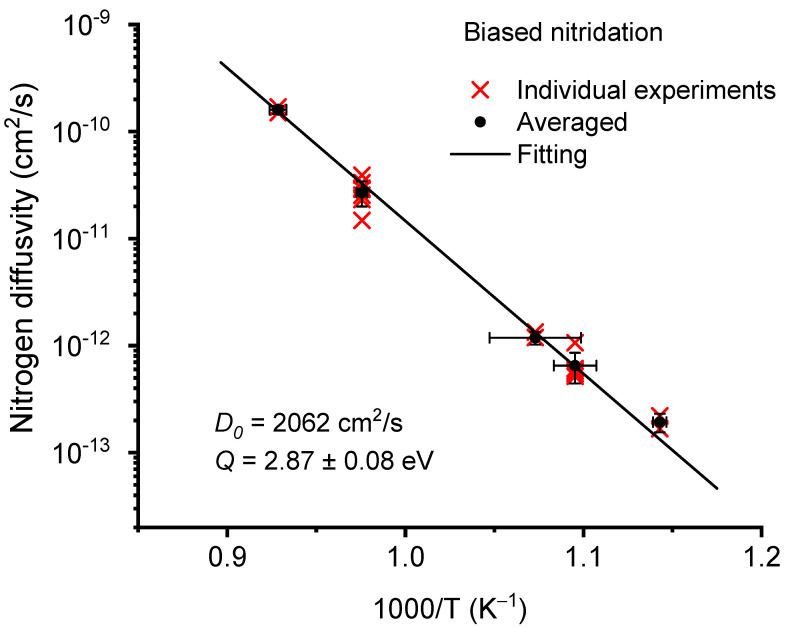
Arrhenius plot for nitrogen diffusivity in Zircaloy-4 as a function of nitridation temperatures for biased nitridation.

Nitridation thermal budgets (temperate and duration) were intentionally selected to be high in the present study to maximize N diffusion. After cross-sectional polishing, the nitrogen diffusion affected layer was clearly visible due to its distinctive hardness changes. The layer thickness was measured under various nitridation conditions, including temperatures from 760 to 840 °C for floating nitridation and from 600 to 800 °C for biased nitridation. The diffusivity was estimated from the layer thickness of the hardened layers according to L =$\;\sqrt{Dt}$, where D is the diffusivity and t is the nitridation time period. Figure 9 plots the Arrhenius plot of nitrogen diffusivities as a function of temperatures using floating CCPN. The Arrhenius equation is $D=D_{0}\exp\left(-\frac{Q}{kT}\right),\;$ where $D_{0}$ is the diffusivity prefactor, Q is the diffusion activation energy, k is the Boltzmann constant, and T is the temperature. The cross symbols refer to results obtained from individual experiments. The square symbols refer to the average values for each temperature point. The error bars represent statistical fluctuation. The extracted diffusivity prefactor is 1708 cm^2^/s and the diffusion activation energy is 2.88 ± 0.05 eV. In comparison, Figure 10 plots N diffusivity for biased CCPN. The diffusivity prefactor is 2062 cm^2^/s and the diffusion activation energy is 2.87 ± 0.08 eV.

## 4. Discussion

**Figure 11:**
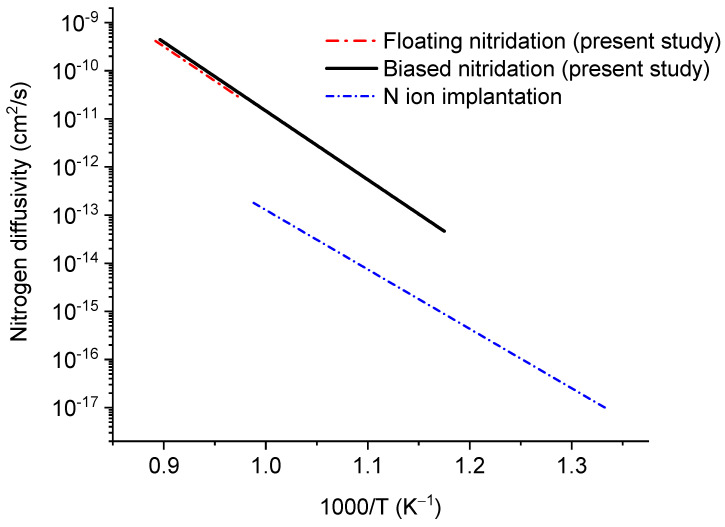
Comparisons of nitrogen diffusivity in Zircaloy-4 for floating nitridation and biased nitridation, both from the present study, and nitrogen diffusivity in α-Zr from a previous nitrogen ion implantation study.

Figure 11 compares both diffusivities and the previous result obtained from the N ion implantation in α-Zr [23]. The N diffusivity in ion-implanted α-Zr, plotted as a dashed line, is significantly lower than the results from the present study. This difference was expected as, in the case of ion irradiation, point defect creation is much more significant, and defect-nitrogen interactions influence nitrogen diffusivity. Vacancies are expected to trap nitrogen atoms and reduce their effective diffusivity. However, there is almost no difference between floating nitridation and biased nitridation. Diffusivity curves from both nitridation methods are close to each other, with almost the same activation energy.

The measured ZrN hardness, peaked at 23 GPa, agrees with previous studies [24]. Due to the sensitivity of hardness to stress, grain sizes, and grain morphology, the measured hardnesses in previous studies ranged from ~15 to ~30 GPa [24,25]. Our nitridation experiments on pure Zr produced a hardness of ~14 GPa [26]. The hardness measured at deep regions beyond nitrogen diffusion layers, in the present study, was about 2–4 GPa. This agrees with previous studies on Zr-1Nb-0.05Cu alloy, which reported 2.7 GPa [27], and the molecular dynamics simulation result, which was about 5 GPa [28].

The present study shows the feasibility of controlling the thickness of the hardened layer by changing nitrogen diffusion by adjusting nitridation temperature and time. Note that our diffusivity was measured from the thickness of the hardened layer. The nitrogen diffusion depth was determined by the critical concentration above which hardening becomes noticeable. Thus, our diffusion length were expected to be larger than the normal diffusion length measured from Gaussian spreading of a buried peak.

In other words, the activation energy here is the same as the diffusion energy barrier, but the measured refactors here might be larger than the values determined using traditional methods. Thermal diffusion of nitrogen in pure zirconium was previously studied by annealing in a nitrogen atmosphere with a temperature gradient [29], or by nitrogen ion implantation [23]. The activation energy of nitrogen in α-Zr was measured to be 2.5 eV, and the frequency prefactor was measured to be 0.56 cm^2^ s^−1^. In comparison, the diffusion activation energy from the present study is about 2.88 eV, and the prefactor is about three orders of magnitudes higher than the previous findings. In addition to the difference in defining diffusion length, there are two other possible reasons for the large difference. One is the difference between Zircaloy-4 and pure Zr, and the other is the sensitivity of diffusivity on the defect population. It was expected that nitrogen diffusion would be assisted through interaction with vacancies. As a non-equilibrium process, plasma nitridation creates supersaturated point defects, so the prefactor was expected to be higher than the equilibrium value.

Nitridation-induced hardening is beneficial to reactor applications. Hardening can increase wear resistance when fuel cladding has close mechanical interactions with other in-core components. Wear of the fuel rod cladding involves the removal of cladding material in an abrasive manner and can lead to the failure of the fuel rod. Grid-to-rod fretting (GTRF) is a flow-induced vibration problem that results in the wear and failure of the rods [30]. As one of the leading causes of nuclear fuel leakage, GTRF wear has cost power utilities millions of dollars in preventive measures. Another wear mechanism is debris fretting, which was a major cause of fuel failures in the 1980s and 1990s [31]. Debris, such as metal particles and chips in the coolant, lodges between the walls of the grid cells and the fuel rods, resulting in abrasive removal of cladding surfaces. This fuel failure mechanism has become less prevalent due to the fuel assembly being fitted with debris filters in the bottom nozzle and improved foreign material exclusion procedures.

Plasma nitridation was previously applied to enhance the wear resistance of AISI 316 stainless steel [32]. The previous works not only showed the effectiveness of nitridation in enhancing the wear resistance of 316 control rod clusters but also demonstrated the feasibility of the nitridation technique to treat tubes of >4 m length with high throughput (>30 tubes in one treatment) [32]. There is no barrier to directly adopting the technique to treat Zircaloy alloys of complicated geometry, from tubes to spacers.

Nitridation treatment can also help to reduce fuel-cladding interactions when fuel swelling closes the fuel-cladding gap. Coating a ZrN thin film on U–Mo powder has been explored using physical vapor deposition and chemical vapor deposition to reduce the interaction of U–Mo fuel with the metal matrix [33,34,35]. Different from other coating methods, plasma nitridation can treat the inner surfaces of hollow fuel cladding tubes.

Enhanced corrosion resistance after nitridation treatment is expected. Both formations of ZrN layer and nitrogen diffusion affected layer are expected to enhance corrosion resistance. ZrN coating is well-known for its high thermal stability and corrosion resistance [36]. For hardened nitrogen diffusion affected layers, the volume expansion due to nitrogen addition will create compressive stress, which will help to delay cracking initiation under corrosive environments.

## 5. Conclusions

We showed the feasibility of using nitrogen plasma treatment to modify Zircaloy-4 surfaces. A thin layer of polycrystalline ZrN forms on the surface, followed by much deeper nitrogen diffusion zones. The structural characterization and mechanical property measurement results are summarized below:

1. The formation of ZrN phase was confirmed by both TEM and Raman spectroscopy.
2. Through a combination of surface indentation and cross-sectional indentation, the study showed that the ZrN layer has a hardness up to 23 GPa, while the nitrogen diffusion zone has a hardness of about 7 to 15 GPa. In comparison, the bulk hardness is about 3 GPa.
3. The hardness-increased zones match the nitrogen profiles.
4. The nitrogen diffusivities were measured under various nitridation conditions. The study showed that a single Arrhenius equation can be used to predict the width changes in nitrogen hardened zones. A diffusion activation energy of 2.88 eV was extracted.

## Data Availability

The data presented in this study are available on request from the corresponding author.
